# Supplementary material for: Consistent variations in personality traits and their potential for genetic improvement in biocontrol agents: Trichogramma evanescens as a case study
Source: Evol Appl. 2022 Feb 11;15(10):1565–79. doi: 10.1111/eva.13329 (PMC9624082; doi:10.1111/eva.13329)
Supplement: Supplementary file 1 — Appendix S1 [file EVA-15-1565-s001.docx]

**Supplementary materials**

Protocol followed to create the lines

The initial populations were established from individuals sampled in different parts of France, from 2010 to 2016, by the INRAE “Egg-Parasitoids Collection” (CRB EP-Coll, Sophia Antipolis) biological resource centre or the company Bioline AgroSciences Ltd. The genetic and phenotypic differences between these populations were not known. As a means of obtaining populations with low levels of genetic variability, we established 14 lines from the initial populations, by brother x sister crossing over 15 generations (Supplementary figure 1.A.). As a means of obtaining lines with mixed genetic backgrounds, we established 10 lines from a mixture of three initial field-sampled populations. We achieved this by mating one virgin female from a first population with one virgin male from a second population. We then selected a virgin female from their offspring and mated it with a virgin male from a third population (Supplementary figure 1.B.). Over the next 13 generations, we performed inbred crosses, as explained above. The experiments described in this study were performed with these 24 lines.


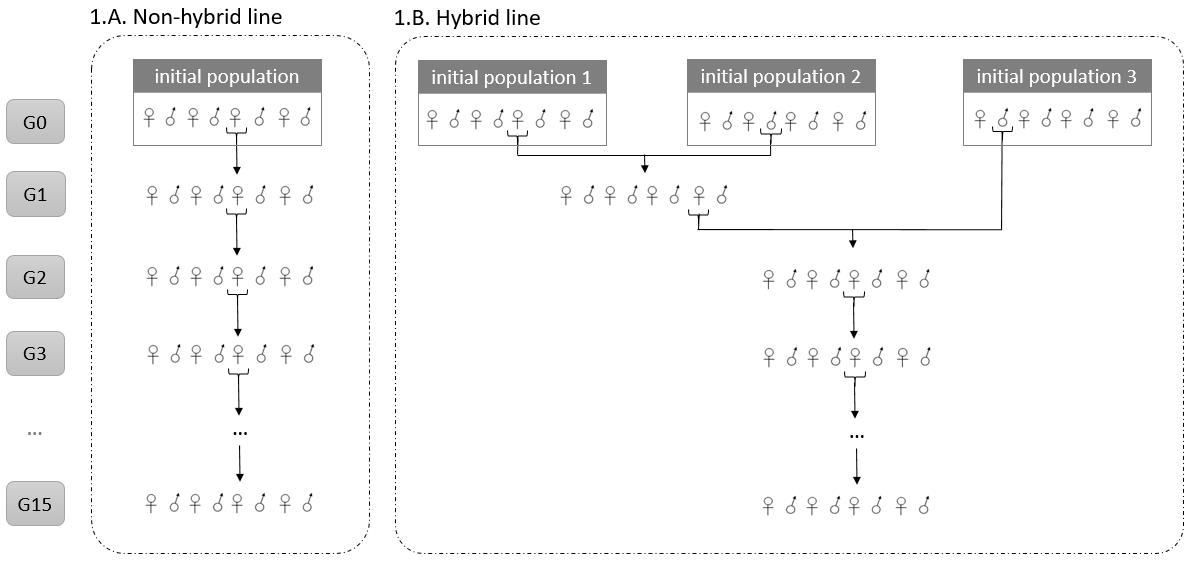


**Supplementary figure 1.** Crossing design for non-hybrid (Suppl. Fig. 1.A.) and hybrid (Suppl. Fig. 1.B.) lines, over 15 generations (G1 to G15).

**Supplementary table 1.** Details on the origins and creation of the lines.

| Line number | Line type | Number of inbred crosses | Initial population (ip) | | Source | Details of capture | | | | | |
| --- | --- | --- | --- | --- | --- | --- | --- | --- | --- | --- | --- |
|  |  |  |  |  |  | **Year** | **Longitude** | **Latitude** | **Altitude** | **Site (county)** | **Host plant name** |
| 1 | Non-hybrid | 15 | A | | CRB | 2015 | 7.36 | 48.20 | 210 | - | *Olea europaea* |
| 2 | Non-hybrid | 15 | B | | CRB | 2015 | 4.96 | 45.52 | 222 | - | *Cydonia oblonga* |
| 3 | Non-hybrid | 15 | D | | CRB | 2016 | 0.89 | 45.20 | 109 | - | *Ulmus sp.* |
| 4 | Non-hybrid | 15 | E | | CRB | 2016 | 0.89 | 45.20 | 109 | - | *Ligustrum sp.* |
| 5 | Non-hybrid | 15 | F | | CRB | 2016 | 0.89 | 45.20 | 109 | - | *Diospyros kaki* |
| 6 | Non-hybrid | 7 | H | | CRB | 2016 | 4.93 | 44.98 | 180 | - | *Malus sp.* |
| 7 | Non-hybrid | 15 | N | | CRB | 2016 | -0.78 | 43.49 | 70 | - | *Solanum lycopersicum* |
| 8 | Non-hybrid | 7 | P | | CRB | 2016 | 6.50 | 43.96 | 899 | - | *Prunus spinosa* |
| 9 | Non-hybrid | 7 | Q | | CRB | 2015 | 3.58 | 45.67 | 628 | - | *Prunus sp.* |
| 10 | Non-hybrid | 7 | BIO_XA | | BIOLINE | 2010 | - | - | - | Saint Jeannet (06) | - |
| 11 | Non-hybrid | 15 | BIO_XE | | BIOLINE | 2013 | - | - | - | Tanneron (83) | - |
| 12 | Non-hybrid | 7 | BIO_XF | | BIOLINE | 2012 | - | - | - | Maleville (12) | - |
| 13 | Non-hybrid | 15 | BIO_XH | | BIOLINE | 2013 | - | - | - | Fourques (30) | - |
| 14 | Non-hybrid | 7 | BIO_XK | | BIOLINE | 2013 | - | - | - | Vernoux (07) | - |
| 15 | Hybrid | 13 | ip 1 | F | CRB | 2016 | 0.89 | 45.20 | 109 | - | *Diospyros kaki* |
|  |  |  | ip 2 | P | CRB | 2016 | 6.50 | 43.96 | 899 | - | *Prunus spinosa* |
|  |  |  | ip 3 | O | CRB |  | 43.49 | -0.78 | 70 | - | *Solanum tuberosum* |
| 16 | Hybrid | 13 | ip 1 | E | CRB | 2016 | 0.89 | 45.20 | 109 | - | *Ligustrum sp.* |
|  |  |  | ip 2 | I | CRB | 2016 | 43.77 | 7.18 | 178 | - | *Vitis sp.* |
|  |  |  | ip 3 | N | CRB | 2016 | -0.78 | 43.49 | 70 | - | *Solanum lycopersicum* |
| 17 | Hybrid | 13 | ip 1 | H | CRB | 2016 | 4.93 | 44.98 | 180 | - | *Malus sp.* |
|  |  |  | ip 2 | A | CRB | 2015 | 7.36 | 48.20 | 210 | - | *Olea europaea* |
|  |  |  | ip 3 | L | CRB |  | 6.94 | 43.61 | S19 | - | *Calicotome spinosa* |
| 18 | Hybrid | 13 | ip 1 | B | CRB | 2015 | 4.96 | 45.52 | 222 | - | *Cydonia oblonga* |
|  |  |  | ip 2 | D | CRB | 2016 | 0.89 | 45.20 | 109 | - | *Ulmus sp.* |
|  |  |  | ip 3 | M | CRB |  | -0.78 | 43.49 | 70 | - | *Olea europaea* |
| 19 | Hybrid | 13 | ip 1 | G | CRB |  | 43.83 | 6.55 | 731 | - | *Calicotome spinosa* |
|  |  |  | ip 2 | Q | CRB | 2015 | 3.58 | 45.67 | 628 | - | *Prunus sp.* |
|  |  |  | ip 3 | K | CRB |  | 6.83 | 43.78 |  | - | *Rosa canina* |
| 20 | Hybrid | 13 | ip 1 | BIO_XB | BIOLINE | 2012 | - | - |  | Cliousclat (26) | - |
|  |  |  | ip 2 | BIO_XL | BIOLINE | 2013 | - | - |  | Douville (24) | - |
|  |  |  | ip 3 | BIO_XH | BIOLINE | 2013 | - | - |  | Fourques (30) | - |
| 21 | Hybrid | 13 | ip 1 | BIO_XE | BIOLINE | 2013 | - | - |  | Tanneron (83) | - |
|  |  |  | ip 2 | BIO_XI | BIOLINE | 2013 | - | - |  | Fourques (30) | - |
|  |  |  | ip 3 | BIO_XK | BIOLINE | 2013 | - | - |  | Vernoux (07) | - |
| 22 | Hybrid | 13 | ip 1 | BIO_XG | BIOLINE | 2012 | - | - |  | Seillans (83) | - |
|  |  |  | ip 2 | BIO_XF | BIOLINE | 2012 | - | - |  | Maleville (12) | - |
|  |  |  | ip 3 | BIO_XB | BIOLINE | 2012 | - | - |  | Cliousclat (26) | - |
| 23 | Hybrid | 13 | ip 1 | BIO_XH | BIOLINE | 2013 | - | - |  | Fourques (30) | - |
|  |  |  | ip 2 | BIO_XC | BIOLINE | 2011 | - | - |  | Mollèges (13) | - |
|  |  |  | ip 3 | BIO_XL | BIOLINE | 2013 | - | - |  | Douville (24) | - |
| 24 | Hybrid | 13 | ip 1 | BIO_XJ | BIOLINE | 2013 | - | - |  | Vernoux (07) | - |
|  |  |  | ip 2 | BIO_XA | BIOLINE | 2010 | - | - |  | Saint Jeannet (06) | - |
|  |  |  | ip 1 | BIO_XI | BIOLINE | 2013 | - | - |  | Fourques (30) | - |
